# Lung abscess complicating a case of multidrug-resistant tuberculosis

**DOI:** 10.11604/pamj.2024.49.3.44889

**Published:** 2024-09-02

**Authors:** Sreeprada Bollineni, Gaurang Aurangabadkar

**Affiliations:** 1 Department of Respiratory Medicine, Jawaharlal Nehru Medical College, Datta Meghe Institute of Higher Education and Research (DMIHER), Deemed to be University, Sawangi (Meghe), Wardha, India,; 2 Department of Respiratory Medicine, Datta Meghe Medical College, Nagpur, Datta Meghe Institute of Higher Education and Research (DMIHER), Deemed to be University, Sawangi (Meghe), Wardha, India

**Keywords:** Multidrug-resistant tuberculosis, lung abscess, bedaquiline

## Image in medicine

A 54-year-old male patient who had a history of pulmonary tuberculosis (TB) 3 years back, and had stopped anti-tubercular therapy after only 2 months of therapy, instead of the recommended WHO guidelines of at least 6 months of therapy. He recently presented with a productive cough with blood-tinged sputum. A chest X-ray posteroanterior (PA) view was done, which revealed the presence of bilateral consolidation with a cavity in the right upper zone with an air-fluid level, suggestive of a lung abscess. The sputum testing of the patient by GeneXpert MTB-Rif technique revealed the detection of *Mycobacterium tuberculosis* (Mtb) with resistance to Rifampicin and Line probe assay (LPA) revealing resistance to Isoniazid. The patient was started on a longer oral bedaquiline-containing multidrug-resistant tuberculosis (MDR-TB) regimen as per Government guidelines after pre-treatment evaluation, along with symptomatic medications. The patient was asked to review after 2 months in the respiratory medicine outpatient department for follow-up. Multidrug-resistant tuberculosis (MDR-TB) is defined by the World Health Organization (WHO) as resistance to Isoniazid and Rifampicin, the two most important and efficacious first-line anti-tubercular drugs. MDR-TB is an exquisitely difficult-to-treat condition requiring a longer duration of treatment ranging from 9 months to 18 months depending upon the spectrum of drug resistance and response to therapy. It is also associated with multiple pulmonary complications such as extensive lung cicatrization, fibrosis, and bronchiectasis amongst other chronic sequelae. A rare sequela found in MDR-TB is associated with lung abscess, which is highlighted in our clinical image.

**Figure 1:**
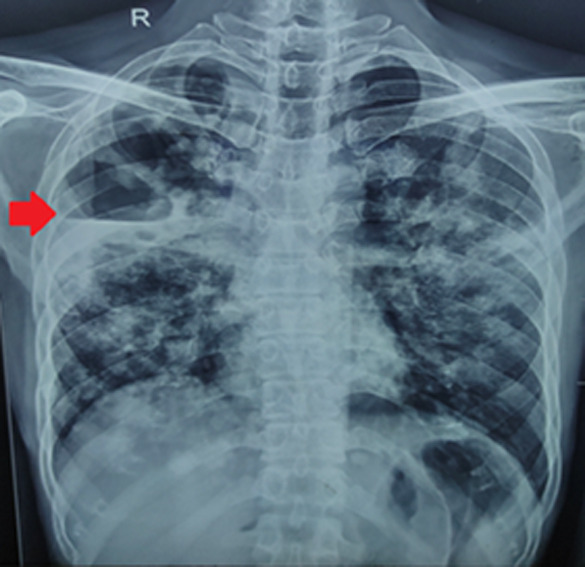
chest X-ray posteroanterior view showing bilateral lung consolidation with a cavitary lesion in the right upper zone (red arrow), with an air-fluid level, suggestive of a lung abscess

